# HSPA12B promotes functional recovery after ischaemic stroke through an eNOS‐dependent mechanism

**DOI:** 10.1111/jcmm.13507

**Published:** 2018-02-07

**Authors:** Yanlin Zhao, Chang Liu, Jiali Liu, Qiuyue Kong, Yu Mao, Hao Cheng, Nan Li, Xioajin Zhang, Chuanfu Li, Yuehua Li, Li Liu, Zhengnian Ding

**Affiliations:** ^1^ Department of Geriatrics Jiangsu Provincial Key Laboratory of Geriatrics The First Affiliated Hospital with Nanjing Medical University Nanjing China; ^2^ Departments of Pharmacology China Pharmaceutical University Nanjing China; ^3^ Departments of Anesthesiology The First Affiliated Hospital with Nanjing Medical University Nanjing China; ^4^ Departments of Surgery East Tennessee State University Johnson City TN USA; ^5^ Department of Pathophysiology Nanjing Medical University Nanjing China; ^6^ Laboratory of Targeted Intervention of Cardiovascular Disease Collaborative Innovation Center for Cardiovascular Disease Translational Medicine Nanjing Medical University Nanjing China

**Keywords:** functional recovery, ischaemic stroke, heat‐shock protein A12B (HSPA12B), angiogenesis, neurogenesis, eNOS

## Abstract

Stroke is the leading cause of disability worldwide. HSPA12B, a heat‐shock protein recently identified expression specifically in endothelial cells, is able to promote angiogenesis. Here, we have investigated its effects on functional recovery at chronic phase of ischaemic stroke. Ischaemic stroke was induced by 60 min. of middle cerebral artery occlusion in transgenic mice with overexpression of HSPA12B (HSPA12B Tg) and wild‐type littermates (WT). HSPA12B Tg mice demonstrated a significant higher survival rate than WT mice within 28 days post‐stroke. Significant improved neurological functions, increased *spontaneous locomotor activity and decreased anxiety were detected in* HSPA12B Tg mice compared with WT controls within 21 days post‐stroke. Stroke‐induced hippocampal degeneration was attenuated in HSPA12B Tg mice examined at day 28 post‐stroke. Interestingly, HSPA12B Tg mice showed enhanced peri‐infarct angiogenesis (examined 28 days post‐stroke) and hippocampal neurogenesis (examined 7 days post‐stroke), respectively, compared to WT mice. The stroke‐induced eNOS phosphorylation and TGF‐β1 expression were augmented in HSPA12B Tg mice. However, administration with eNOS inhibitor L‐NAME diminished the HSPA12B‐induced protection in neurological functional recovery and mice survival post‐stroke. The data suggest that HSPA12B promoted functional recovery and survival after stroke in an eNOS‐dependent mechanism. Targeting HSPA12B expression may have a therapeutic potential for the stroke‐evoked functional disability and mortality.

## Introduction

Stroke occurs in someone every 40 sec. in the United States 1. Improvements in acute stroke patient care result in reduced stroke mortality whereas leaves more survivors with severe disability, which makes stroke the leading cause of disability worldwide 2, 3. However, identification of therapeutic targets for promoting functional recovery in chronic stroke is largely limited.

Angiogenesis and neurogenesis present in the brains of stroke patients 4. Angiogenesis, defined as new microvessel formation *via* branching off from pre‐existing vessels, is positively correlated with the outcome of stroke patients 5. Angiogenesis after stroke not only replenishes blood flow to the ischaemic area, but also promotes neurogenesis and improves neurological functions in both animal models and patients 5, 6, 7. Therefore, promoting angiogenesis represents a promising therapeutic target for improving functional recovery in chronic stroke patients.

Heat‐shock protein A12B (HSPA12B) is expressed specifically in endothelial cells 8, 9. Studies demonstrate that HSPA12B is up‐regulated during angiogenesis and plays essential roles in proliferation and migration of endothelial cells *in vitro* and vascular development in zebrafish *in vivo* 8, 9. Using transgenic mice with overexpression of HSPA12B (HSPA12B Tg), we revealed that HSPA12B protects hearts from myocardial infarction, ischaemia/reperfusion injury and endotoxin challenge 10, 11, 12. Of particular interest to this study, we reported recently that HSPA12B attenuated neuronal apoptosis at the acute phase (within 24 hrs) of ischaemic stroke 13. However, whether HSPA12B plays a role in neurological functional recovery in chronic stroke is unknown.

To answer this question, we examined neurological functions at chronic phase (21 days) of ischaemic stroke. We observed that HSPA12B Tg mice have promoted functional recovery and increased survival post‐stroke, concomitant with increased angiogenesis and neurogenesis. We provide evidence that activation of eNOS is responsible for the promotion of functional recovery by HSPA12B in chronic stroke.

## Materials and methods

### Antibodies and chemicals

N_ω_‐Nitro‐l‐arginine methyl ester hydrochloride (L‐NAME), primary antibodies for α smooth muscle actin (α‐SMA), BrdU and GAPDH were purchased from Sigma‐Aldrich (St Louis, MO, USA). Primary antibody for HSPA12B was a generous gift from Dr. Zhihua Han 9. Primary antibodies for TGF‐β1 and TGF‐β2 were from Bioworld (Louis Park, MN, USA). Primary antibodies for CD31, BDNF, eNOS, phosphor‐eNOS (p‐eNOS) and NeuN were from BD Pharmingen (San Jose, CA, USA), Santa Cruz (Dallas, TX), Abcam (Cambridge, MA, USA), Cell Signaling Technology (Beverly, MA, USA) and Millipore (Temecula, CA, USA), respectively. The Supersignal West Pico Chemiluminescent Substrate was obtained from Pierce (Rockford, IL, USA).

### Animals

HSPA12B Tg mice (C57/BL6 strain) were generated as described in our previous studies 11. Mice were housed in the Animal Laboratory Resource Facility, Model Animal Research Center (MARC) of Nanjing University. Mice were fed standard chow diet and tap water *ad libitum* and housed under controlled temperature (22°C) and 12‐h dark–light cycles. All of the experiments were performed with the guidelines for the “Principles of Laboratory Animal Care” and the “Guide for the care and use of laboratory animals” published by the NIH (NIH Publication, 8^th^ Edition, 2011). The animal ethics and experimental protocols were approved by the Nanjing University Committee on Animal Care.

### Ischaemic stroke

Ischaemic stroke was induced by transient middle cerebral artery occlusion for 60 min. followed by reperfusion for the indicated times 13, 14, 15. Briefly, male HSPA12B Tg and wild‐type (WT) littermates weighing 24–26 g at the age of 8–10 weeks were anesthetized by the inhalation of 1.5–2% isoflurane. A 6‐0 filament coated with silicon resin (Doccol Corp. Sharon, MA) was introduced into the left common carotid artery and advanced 11 mm to begin ischaemia. Reperfusion was achieved by removing the filament after 60 min. of occlusion. The successful ischaemic surgery was verified and recorded by the measurement of cerebral blood flow with a laser Doppler flowmetry (BPM2 System, Vasamedics Inc., St. Paul, MN, USA) as described in our previously studies 13. Body temperature was maintained throughout the procedure, from the beginning of the surgery until palinesthesia was observed, at 36.5–37.0°C by means of a lamp and a heating blanket. Sham‐operated mice served as controls. The examinations of infarct volume, histological analysis and quantification, survival recording, neurological scoring and behavioural tests were performed by the trained investigators who were blinded to the genotypes and treatment. The following mice were excluded from the experiment: No appropriate anaesthesia was obtained within 5 min. after inhalation with isoflurane, body temperature during surgery was out of a range of 36.0–37.5°C, bleeding more than 75 μl, the lack of a robust neurological deficit after surgery, no efficient MCA blood and reperfusion; died during or immediately after surgery due to intracranial haemorrhage, the mice with extreme large or small infarct lesion within group.

In eNOS inhibition experiments, mice were treated with eNOS inhibitor L‐NAME by intraperitoneal injection 30 min. prior ischaemia (15 mg/kg) and after ischaemia (5 mg/kg/3 days) as described previously 12, 16.

### Mice survival

Mice survival was recorded twice each day for 28 days after ischaemic stroke.

### Functional outcome tests

All the following experiments were measured at 21 days after stroke by a trained observer who was blinded to the genotypes and treatments of the mice.

#### Neurological evaluation

The animals were evaluated from the next day to 21 days post‐stroke. Neurological function was scored by five different neurological tests: (*i*) spontaneous activity of movement, (*ii*) symmetry of movement, (*iii*) floor walking, (*iv*) bean walking and (*v*) response to vibrissae touch of right side. The scoring system ranged from 0 to 15, in which 15 is a perfect score and 0 is death due to cerebral ischaemic stroke 13. Mice before stroke surgery received a score of 15.

#### Open‐field test

Open‐field measures spontaneous locomotor activity in a novel environment and has been used in stroke models 17, 18. The mice were placed in the centre of a square open‐field chamber (50 cm long × 50 cm wide × 40 cm high) surrounded by walls. The total length of moving path and the averaged moving speed were measured over the course of 20 min. using an automatic monitoring system (O'Hara & Co., Ltd., Tokyo, Japan).

#### Elevated plus maze

Elevated plus maze is a test that assesses anxiety‐like behaviours and ambulatory activities 18, 19. The elevated plus maze (EP‐3002; O'Hara & Co., Ltd.) consisted of two open arms (25 × 5 cm) and two enclosed arms of the same size extending from a central area (5 × 5 cm) and elevated 50 cm from the ground. Mice were placed in the central square of the maze facing one of the open arms. Mouse behaviour was recorded during a 10‐min. test period by means of a Macintosh computer using Image OFCR 1.00× and Image OF circle 1.01× (O'Hara & Co., Ltd.), a modified software based on the public domain of NIH Image program. The frequency of entries into open and closed arms was recorded.

### Histological analysis

The ischaemic hemispheres were collected at 28 days post‐stroke. Paraffin‐embedded sections were prepared and subjected to haematoxylin–eosin (HE) staining to evaluate the histological changes in the hippocampus.

### Immunofluorescence staining

The ischaemic hemispheres were collected for immunofluorescence staining according to our previous methods 10. Briefly, brain tissues at hippocampus levels were collected and processed for cryosectioning at 4 μm. After blocking with 7.5% normal goat serum for 1 hr, the cryosections were incubated with the indicated primary antibodies overnight at 4°C. After thoroughly washing, Cy3‐ or FITC‐conjugated appropriate secondary antibodies were added to the sections to visualize the staining. Hoechst 33342 reagent was used to counterstain the nuclei. The staining was observed using a fluorescence microscope at a magnification of 400× (Olympus, Tokyo, Japan).

### Angiogenesis

The ischaemic hemispheres were collected 28 days post‐stroke for immunofluorescence staining against CD31, a marker of endothelial cells, and α‐SMA, a marker of vessel smooth muscle cells 20. The counts and areas of capillaries (CD31) and arterioles (α‐SMA) were measured in five to ten randomly selected areas of peri‐infarct areas in each sample using a computerized software (Olympus).

### Neurogenesis

BrdU was administrated intraperitoneally (100 mg/kg/day) for 7 days post‐stroke. The ischaemic hemispheres were collected at 7 days post‐stroke for co‐immunofluorescence staining against BrdU and NeuN, a marker of neurons. The cells positive with BrdU and cells positive with BrdU/NeuN were measured in ischaemic hippocampus in each sample using computerized software (Olympus).

### Immunoblotting analysis

The ischaemic hemispheres were collected at day 7 post‐stroke unless stated elsewhere. The protein extracts were prepared for immunoblotting analysis according to the methods described in our previous studies 10, 13. Briefly, equal amount of proteins were prepared, separated on 10% SDS‐PAGE and transferred onto Immobilon‐P membranes (Millipore). The membranes were probed with appropriate primary antibodies followed by incubation with peroxidase‐conjugated secondary antibodies. The signals were detected by enhanced Pierce chemiluminescence. The blots against GAPDH served as loading controls. The signals were quantified by scanning densitometry, and the results from each experimental group were expressed as relative integrated intensity compared with that of controls.

### Statistical analysis

Results are expressed as mean ± standard deviation (*X* ± S.D.). Comparison analysis between groups was performed using a two‐way analysis of variance. Tukey's procedure for multiple range tests was performed. *P <* 0.05 was considered to be significant.

## Results

### Prolonged up‐regulation of HSPA12B in ischaemic brain tissues post‐stroke

**Figure 1:**
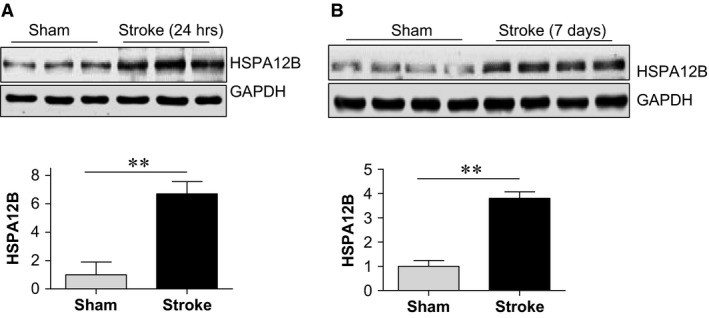
Prolonged up‐regulation of HSPA12B after ischaemic stroke. Ischaemic hemispheres were collected at 24 hrs (**A**) or 7 days (**B**) after ischaemic stroke. Protein extracts were prepared for immunoblotting analysis against HSPA12B. The blots against GAPDH served as loading controls. Data are expressed as mean ± S.D. **P < 0.01, *n* = 3 per group (**A**) and *n* = 4 per group (**B**).

To investigate a possible involvement of HSPA12B in the functional recovery post‐stroke, HSPA12B expression was examined. Immunoblotting analysis demonstrated that HSPA12B expression was increased significantly by 5.7‐fold 24 hrs post‐stroke compared to sham controls (*P* < 0.01; Fig. 1A). The increase in HSPA12B expression persisted to 7 days post‐stroke (*P* < 0.01; Fig. 1B). Thus, HSPA12B was up‐regulated in a prolonged manner after ischaemic stroke.

### HSPA12B overexpression improves mice survival at chronic phase of stroke

**Figure 2:**
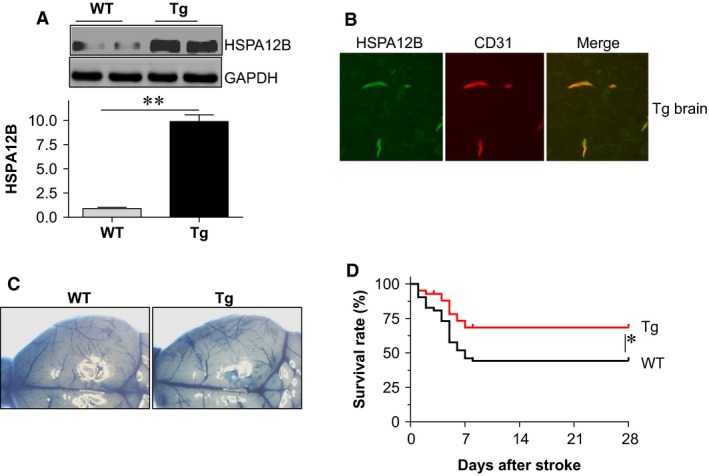
HSPA12B Tg mice showed improved survival after ischaemic stroke. (**A**) HSPA12B expression. Ventricular tissues were collected from WT and HSPA12B Tg mice (8 weeks old) for immunoblotting analysis against HSPA12B. The blots against GAPDH served as loading controls. **P < 0.01, *n* = 4 per group. (**B**) HSPA12B localization. Brain tissues collected from HSPA12B Tg mice were prepared for immunostaining against HSPA12B and CD31. Note that HSPA12B (green) was colocalized with CD31 (red), a marker of endothelial cells. The representative images were from three independent mice. (**C**) Gross anatomy of the middle cerebral artery territory. Adult HSPA12B Tg and WT mice were perfused with Evans blue through intravenous injection. Images were captured under stereomicroscope. (**D**) Survival. Mice survival was recorded twice a day within 28 days after stroke. *P < 0.05, *n* = 52 for WT group and *n* = 41 for Tg group.

We then evaluated the roles of up‐regulated HSPA12B in mice survival at chronic phase of stroke using HSPA12B Tg mice. The overexpression of HSPA12B in cerebral endothelial cells of Tg mice was confirmed by immunoblotting analysis and immunofluorescence staining for HSPA12B (Fig. 2A and B). Two genotypes showed comparable gross anatomy of the middle cerebral artery territory (Fig. 2C), as indicated by blood vessels stained with Evans blue 21. As expected, HSPA12B Tg mice demonstrated a significant higher survival rate than WT mice did within 28 days post‐stroke (*P* < 0.05; Fig. 2D).

### HSPA12B overexpression accelerates functional recovery at chronic phase of ischaemic stroke

**Figure 3:**
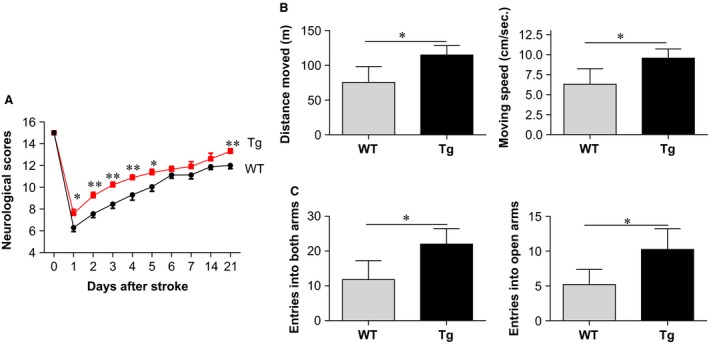
HSPA12B Tg mice showed improved functional recovery after ischaemic stroke. (**A**) Neurological scoring. The neurological scores were evaluated every day from the day before stroke to 21 days after stroke. The scoring system ranged from 0 to 15, in which 15 represented a perfect score and 0 represented death due to stroke injury. **P < 0.01 or *P < 0.05 *versus* time‐matched WT mice. *n* = 38 for Tg group, *n* = 29 for WT group. (**B**) Open‐field test. This test was used to evaluate the *spontaneous locomotor activity* in mice 21 days post‐stroke. The total distance moved in 20 min. was recorded, and the averaged movement speed was calculated. *P < 0.05, *n* = 5 per group. (**C**) Elevated plus maze test. This test was used to evaluate *locomotor activity* and anxiety in mice 21 days post‐stroke. The entries into open and closed arms were recorded in 10 min. *P < 0.05, *n* = 4–5 per group.

The neurological function was evaluated from the day before stroke to 21 days post‐stroke using neurological scoring, which consisting of spontaneous activity of movement, symmetry of movement, floor walking, bean walking and response to vibrissae touch of right side. The scores obtained 24 hrs prior to ischaemic insult served as baseline controls. As shown in Figure 3A, ischaemic stroke decreased neurological scores in both WT and Tg mice, respectively, compared with their baseline controls. The lowest scores were detected at 24 hrs post‐stroke and were recovered gradually after then in both genotypes. However, HSPA12B Tg mice exhibited significant higher scores than those in the time‐matched WT mice post‐stroke (*P* < 0.01 or 0.05), respectively.

Spontaneous motor function was also evaluated using open‐field test at day 21 post‐stroke (Fig. 3B). HSPA12B Tg mice demonstrated 52.3% more moving distance within 20 min. compared to WT mice (*P* < 0.05). Also, a significant faster moving speed (51.7%) was detected in HSPA12B Tg mice compared with WT mice (*P* < 0.05).

Finally, elevated plus maze was used to test anxiety and motor activity in mice at day 21 post‐stroke 22, 23. Significant more (86.4%) total entries into both open and closed arms were recorded in HSPA12B Tg mice compared to WT mice (*P* < 0.05; Fig. 3C). Notably, HSPA12B Tg mice demonstrated more entries (97.1%) into open arms than WT mice did, suggesting a decreased anxiety in HSPA12B Tg mice post‐stroke.

### HSPA12B overexpression attenuates hippocampal degeneration at chronic phase of stroke

**Figure 4:**
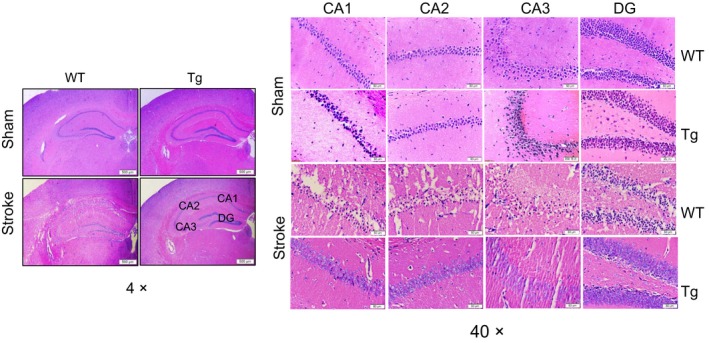
HSPA12B Tg mice showed attenuated hippocampal degeneration after ischaemic stroke. Ischaemic hemispheres were collected 28 days after stroke. The results of HE staining on paraffin‐embedded sections showed an attenuated neuronal morphological abnormalities in HSPA12B Tg mice. The boxed areas of CA1, CA2, CA3 and dentate gyrus (DG) were magnified in the right panels. The representative images were from three independent experiments. Scale bar = 500 μm for left panel and scale bar = 50 μm for right panel.

Morphological alterations in hippocampal CA1, CA2, CA3 and dentate gyrus fields were assessed by H&E staining. The results showed that hippocampal neurons in sham mice exhibited round and pale‐blue stained, which is typical of healthy cells (Fig. 4). At day 28 post‐stroke, WT mice demonstrated neuronal shrinkage and nuclear condensation. However, the stroke‐induced abnormalities of neuronal morphology in all fields of hippocampus were significantly attenuated in HSPA12B Tg mice, respectively, compared with that in WT mice.

### HSPA12B overexpression promotes peri‐infarct angiogenesis post‐stroke

**Figure 5:**
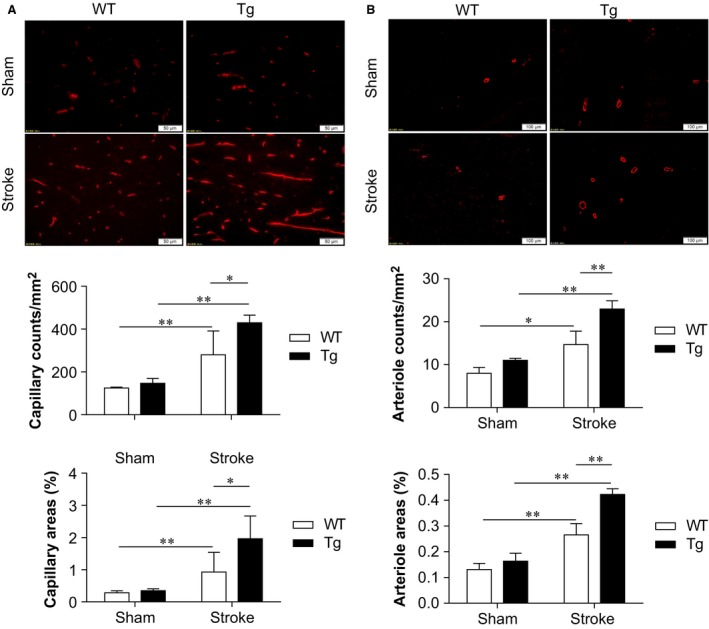
HSPA12B Tg mice showed increased angiogenesis after ischaemic stroke. Ischaemic hemispheres were collected 28 days after stroke. The cryosections were prepared for immunostaining against CD31 (**A**) and α‐SMA (**B**). The angiogenesis in peri‐infarct areas was evaluated by the counts and areas of capillaries and arterioles, respectively. **P < 0.01 or *P < 0.05, *n* = 3–6 per group. Scale bar = 50 μm for capillary staining and scale bar = 100 μm for arteriole staining.

Angiogenesis has been shown to be critical in improving post‐stroke neurological functional recovery 4. We therefore examined angiogenesis by immunofluorescence staining against CD31 (a marker of endothelial cells) and α‐SMA (a marker of smooth muscle cells of vessels) in peri‐infarct regions at day 28 post‐stroke. As shown in Figure 5A, stroke increased capillary counts and areas in both HSPA12B Tg and WT mice, respectively, compared with their sham controls (*P* < 0.01). Notably, the stroke‐induced increases of capillary counts and areas were enhanced in HSPA12B Tg mice by 53.3% and 99.8%, respectively, compared with WT mice (*P* < 0.05).

Figure 5B shows that stroke also increased arteriole counts and areas in both HSPA12B Tg and WT mice, respectively, compared with their sham controls (*P* < 0.01 or 0.05). However, HSPA12B Tg mice demonstrated significant more arteriole counts and areas by 56.3% and 236.0%, respectively, compared with WT mice post‐stroke (*P* < 0.01 or 0.05).

### HSPA12B overexpression promotes neurogenesis in hippocampus post‐stroke

**Figure 6:**
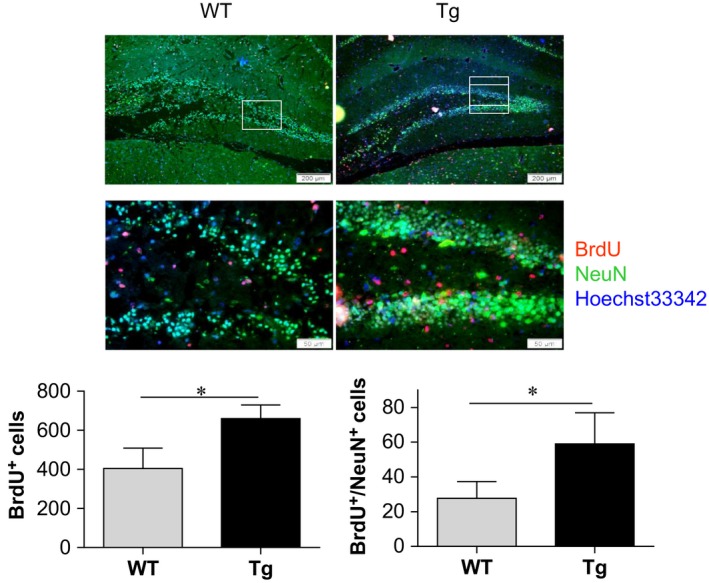
HSPA12B Tg mice showed enhanced neurogenesis after ischaemic stroke. Ischaemic hemispheres were collected from mice that administrated with BrdU for 7 days after stroke. The paraffin‐embedded sections were prepared for immunostaining against BrdU and NeuN. Hoechst 33342 was used to counterstain nuclei. The cells stained positively with BrdU (BrdU^+^) and double positively with BrdU and NeuN (BrdU^+^/NeuN^+^) in hippocampus were measured using a fluorescence microscope. The boxed areas were magnified in down panel. *P < 0.05, *n* = 3–4 per group. Scale bar = 200 μm for up panel and scale bar = 50 μm for down panel.

Neurogenesis has shown a positive relationship with the functional recovery after stroke. Thus, neurogenesis was examined in hippocampus using BrdU incorporation at day 7 post‐stroke. As shown Figure 6, HSPA12B Tg mice exhibited a significant more BrdU‐positive (BrdU^+^) cells by 63.2% than WT mice did post‐stroke (*P* < 0.05). Importantly, 113.3% more BrdU^+^ cells were co‐stained with NeuN in HSPA12B Tg mice compared to WT mice, indicating a promoted neurogenesis in HSPA12B Tg mice after stroke.

### HSPA12B overexpression enhanced eNOS activation in ischaemic hemispheres

**Figure 7:**
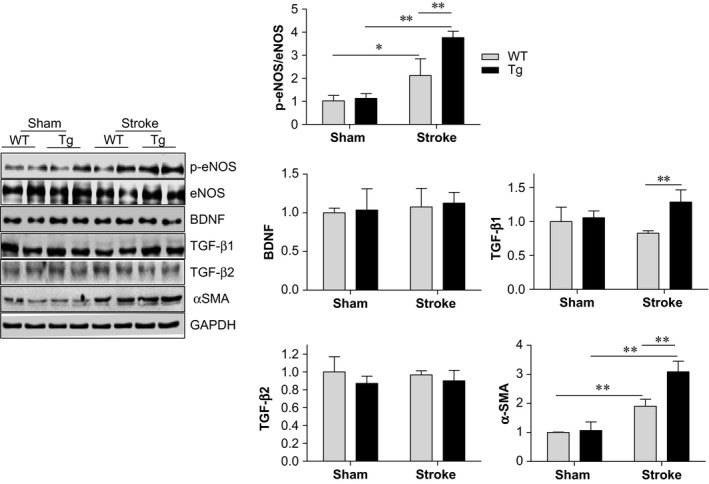
HSPA12B Tg mice showed augmented eNOS activation after ischaemic stroke. Ischaemic hemispheres were collected 7 days post‐stroke. Protein extracts were prepared for immunoblotting analysis against the indicated proteins. The blots against GAPDH served as loading controls. **P < 0.01 or *P < 0.05, *n* = 4 per group.

Activation of eNOS‐dependent signalling has been shown playing roles in both angiogenesis and neurogenesis after stroke 4, 24. Immunoblotting analysis revealed that p‐eNOS/eNOS ratios were increased significantly in ischaemic hemispheres of WT and HSPA12B Tg mice at day 7 post‐stroke, respectively, compared with their sham controls (*P* < 0.01 or 0.05; Fig. 7). Notably, the stroke‐induced increase in p‐eNOS/eNOS ratio was enhanced by 77.7% in HSPA12B Tg mice compared with WT mice (*P* < 0.01). No significant changes were observed in BDNF, one of the downstream targets of eNOS, between genotypes either in sham or in stroke groups.

TGF‐β has been demonstrated to be involved in the eNOS‐mediated angiogenesis as well as in neurogenesis 25, 26, 27. Interestingly, HSPA12B Tg mice showed significant higher levels of TGF‐β1 by 55.7% than those in WT mice post‐stroke (*P* < 0.01; Fig. 7). TGF‐β2 showed no significant changes between genotypes in either sham or stroke groups.

In consistent with the immunostaining results in Figrue 5B, α‐SMA expression was significantly increased in both HSPA12B Tg and WT mice, respectively, compared with their sham controls (*P* < 0.01; Fig. 7). However, the stroke‐induced up‐regulation of α‐SMA was enhanced in HSPA12B Tg mice compared with WT mice (*P* < 0.01).

### Inhibition of eNOS with L‐NAME diminished HSPA12B‐induced improvement of mice survival at chronic phase of stroke

**Figure 8:**
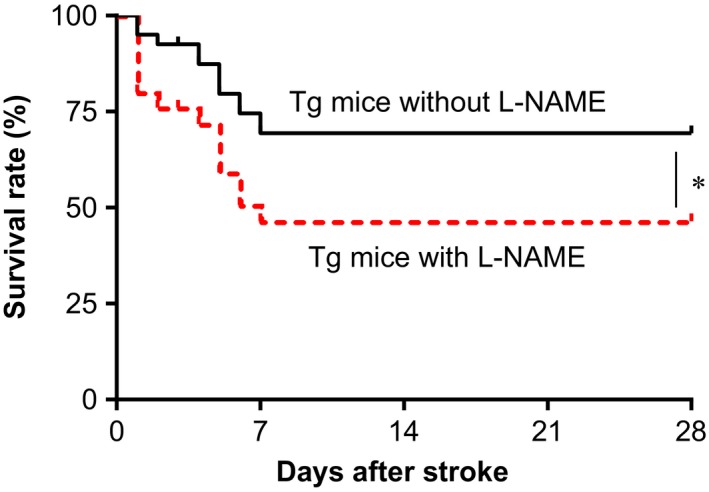
Administration with eNOS inhibitor diminished survival protection in HSPA12B Tg mice after ischaemic stroke. HSPA12B Tg mice were administrated with eNOS inhibitor L‐NAME after stroke. Mice survival was recorded twice a day within 28 days after stroke. *P < 0.05 *versus* time‐matched Tg mice subjected to stroke following L‐NAME administration. *n* = 24 for Tg group with L‐NAME administration and *n* = 39 for Tg group without L‐NAME treatment.

To clarify the roles of eNOS activation in HSPA12B‐induced neuronal protection at chronic phase of stroke, we treated mice with L‐NAME, a widely used eNOS inhibitor in HSPA12B Tg mice 28. Interestingly, administration with L‐NAME significantly decreased mice survival within 28 days post‐stroke in HSPA12B Tg mice compared with HSPA12B Tg mice that without L‐NAME administration (*P* < 0.05; Fig. 8).

### Inhibition of eNOS with L‐NAMA decreased HSPA12B‐induced improvement of neurological functions at chronic phase of stroke

**Figure 9:**
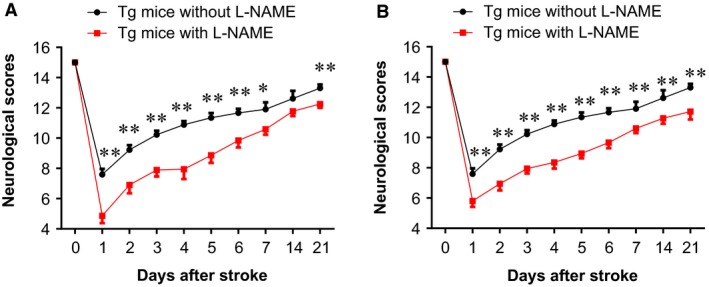
Administration with eNOS inhibitor diminished neurological protection in HSPA12B Tg mice after ischaemic stroke. HSPA12B Tg mice were administrated with eNOS inhibitor L‐NAME prior to (**A**) or immediately after (**B**) stroke. Neurological function was measured daily within 21 days after stroke. The values obtained 24 hrs prior to stroke insult served as baseline controls. **P < 0.01 *versus* time‐matched Tg mice subjected to stroke following L‐NAME administration. *n* = 22 for Tg group with L‐NAME pre‐administration, *n* = 20 for Tg group with L‐NAME post‐administration and *n* = 29 for Tg group without L‐NAME treatment.

Neurological function was also evaluated in HSPA12B Tg mice post‐stroke following L‐NAME administration. As shown in Figure 9A, pre‐administration with L‐NAME decreased neurological scores significantly in HSPA12B Tg mice within 21 days post‐stroke compared with the time‐matched HSPA12B Tg mice without L‐NAME administration (*P* < 0.01 or 0.05), respectively. Also, a significant delayed neurological recovery was observed in HSPA12B‐Tg mice that administrated with L‐NAME immediately after stroke (*P* < 0.01; Fig. 9B).

### Inhibition of eNOS with L‐NAMA decreased HSPA12B‐induced angiogenesis and neuronal cell proliferation at chronic phase of stroke

**Figure 10:**
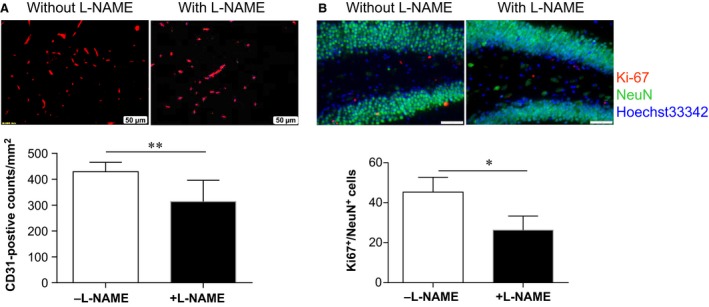
Administration with eNOS inhibitor diminished the promotion of angiogenesis and neuronal cell proliferation in HSPA12B Tg mice after ischaemic stroke. HSPA12B Tg mice were administrated with eNOS inhibitor L‐NAME prior to stroke. Paraffin‐embedded sections were prepared at 28 days post‐stroke. Immunostaining for CD31 (**A**) and Ki‐67 with NeuN (**B**) was performed. Hoechst 33342 was used to counterstain nuclei. **P < 0.01 or *P < 0.05. *n* = 6 (CD31) or 3 (Ki‐67) for Tg group without L‐NAME administration, *n* = 3 for Tg group with L‐NAME administration. Scale bar = 50 μm.

In line with the effect of L‐NAME on neurological recovery in HSPA12B Tg mice after stroke, L‐NAME significantly reduced capillary counts as indicated by immunostaining for CD31 at 28 days post‐stroke (Fig. 10A). Also, L‐NAME significantly reduced neuronal cell proliferation as indicated by immunostaining for Ki‐67 and NeuN at 28 days post‐stroke (Fig. 10B).

## Discussion

We demonstrate for the first time that overexpression of HSPA12B promoted neurological function recovery and improved survival at chronic phase of ischaemic stroke. In addition, a role of eNOS has been identified in regulating functional recovery in HSPA12B Tg mice post‐stroke. The data suggest that targeting HSPA12B expression could serve as a potential target for the management of functional disability after ischaemic stroke.

Stroke affects 15 million people globally, with one‐third of the affected population having permanent disability, impacting quality of life 29, 30. In this study, we revealed that ischaemic stroke up‐regulated HSPA12B expression and this up‐regulation prolonged at least to 7 days post‐stroke. Importantly, overexpression of HSPA12B improved recovery of spontaneous movement activity (including speed and distance), symmetry of movement, floor walking, bean walking and response to vibrissae touch after stroke. Moreover, overexpression of HSPA12B decreased stroke‐induced anxiety. The results suggest that HSPA12B promotes functional recovery after stroke.

It is now appreciated that emerging therapeutic strategies for recovery should include the cerebral vasculature and that induction of angiogenesis will stimulate endogenous recovery mechanisms including neurogenesis, synaptogenesis, and neuronal and synaptic plasticity 30, 31. As examples, angiogenesis is involved in the promotion of post‐stroke functional recovery by non‐pharmacologic interventions (*e.g*. exercise preconditioning and low‐level laser therapy) and pharmacologic interventions (*e.g*. statins and growth factor) 31. On the other hand, impeded post‐stroke recovery was accompanied by impaired reparative angiogenesis in type 2 diabetic rats 30. In this study, we observed an increased angiogenesis in HSPA12B Tg mice after stroke, suggesting facilitating angiogenesis could contribute to the promotion of functional recovery by HSPA12B.

Stroke causes cell death but also birth and migration of new neurons (neurogenesis) within sites of ischaemic damage 32. To date, a fair amount of literature demonstrated a coupling of angiogenesis and neurogenesis post‐stroke 4, 31, 33. The processes of neurogenesis and angiogenesis after stroke are linked together and co‐ordinated. In this study, we demonstrated that overexpression of HSPA12B increased both BrdU‐positive and BrdU/NeuN‐positive cells in hippocampus after stroke, suggesting HSPA12B promoted neurogenesis in stroke brains.

Endothelial nitric oxide synthase (eNOS) plays critical roles in both angiogenesis and neurogenesis after stroke 24. Increased eNOS phosphorylation has been shown mediating the effects of synthetic liver X receptor agonist on the promotion of angiogenesis and vascular maturation, and improvement of functional outcome after stroke 34. On the other hand, eNOS knockout mice exhibited decreased subventricular zone progenitor cell proliferation and migration, and decreased angiogenesis in ischaemic brains. eNOS may regulate angiogenesis through BDNF, TGF‐β1 and other growth factors 24, 35, 36. It is interesting that acute administration with nicotine, a component of cigarette smoke, has been shown to stimulate activity of recombinant eNOS or eNOS isolated from endothelial cells in an *in vitro* experiment 37. By striking contrast, Wang *et al*. 38 reported that chronic nicotine treatment impairs the restoration of blood flow, worsens the neurologic outcome, and enhances brain injury following an ischaemic insult. The modulation of eNOS activity by nicotine may be affected by the duration of nicotine treatment. More recently, HSPA12B has been to act indirectly *via* thioredoxin with subsequent increased expression of VEGF that promotes angiogenesis 39. We have reported that HSPA12B protected acute ischaemia/reperfusion injuries in brain and heart through activation of PI3K/Akt signalling 10, 13. Here, we demonstrated that in the late phase of ischaemic stroke, HSPA12B improved functional recovery through an eNOS‐dependent mechanism. Previous studies revealed that heat‐shock proteins, such as HSP90 can activate AMPK, thereby increasing eNOS activity 40, 41. Indeed, we revealed that the promoted angiogenesis and neurogenesis were concomitant with the enhanced eNOS activation and TGF‐β1 expression in HSPA12B Tg mice post‐stroke. In supporting this, we reported previously that HSPA12B promotes angiogenesis in ischaemic heart through eNOS activation 12. Importantly, administration with eNOS inhibitor L‐NAME diminished the protection of HSPA12B in mice survival and neurologic function recovery post‐stroke, suggesting that eNOS‐mediated neuroprotective effects of HSPA12B at chronic phase of ischaemic stroke.

In summary, HSPA12B overexpression promoted mice survival and functional recovery at chronic phase of ischaemic stroke. The protective action of HSPA12B was mediated, at least in part, through an eNOS‐dependent mechanism.

## Conflict of interest

The authors declare no conflict of interests.

## References

[1] Benjamin EJ , Blaha MJ , Chiuve SE , *et al* Heart disease and stroke statistics‐2017 update: a report from the American Heart Association. Circulation. 2017; 135: 2252–2262. 28122885 10.1161/CIR.0000000000000485 PMC5408160

[2] Kim E , Woo MS , Qin L , *et al* Daidzein augments cholesterol homeostasis *via* ApoE to promote functional recovery in chronic stroke. J Neurosci. 2015; 35: 15113–26. 26558782 10.1523/JNEUROSCI.2890-15.2015 PMC4642242

[3] Loubinoux I , Kronenberg G , Endres M , *et al* Post‐stroke depression: mechanisms, translation and therapy. J Cell Mol Med. 2012; 16: 1961–9. 22348642 10.1111/j.1582-4934.2012.01555.x PMC3822966

[4] Ruan L , Wang B , ZhuGe Q , *et al* Coupling of neurogenesis and angiogenesis after ischemic stroke. Brain Res. 2015; 1623: 166–73. 25736182 10.1016/j.brainres.2015.02.042 PMC4552615

[5] Seto SW , Chang D , Jenkins A , *et al* Angiogenesis in ischemic stroke and angiogenic effects of Chinese herbal medicine. J Clin Med. 2016; 5: E56. 27275837 10.3390/jcm5060056 PMC4929411

[6] Krupinski J , Kaluza J , Kumar P , *et al* Role of angiogenesis in patients with cerebral ischemic stroke. Stroke. 1994; 25: 1794–8. 7521076 10.1161/01.str.25.9.1794

[7] Arenillas JF , Sobrino T , Castillo J , *et al* The role of angiogenesis in damage and recovery from ischemic stroke. Curr Treat Options Cardiovasc Med. 2007; 9: 205–12. 17601384 10.1007/s11936-007-0014-5

[8] Hu G , Tang J , Zhang B , *et al* A novel endothelial‐specific heat shock protein HspA12B is required in both zebrafish development and endothelial functions *in vitro* . J Cell Sci. 2006; 119: 4117–26. 16968741 10.1242/jcs.03179

[9] Steagall RJ , Rusinol AE , Truong QA , *et al* HSPA12B is predominantly expressed in endothelial cells and required for angiogenesis. Arterioscler Thromb Vasc Biol. 2006; 26: 2012–8. 16825593 10.1161/01.ATV.0000235720.61091.c7

[10] Kong Q , Dai L , Wang Y , *et al* HSPA12B attenuated acute myocardial ischemia/reperfusion injury *via* maintaining endothelial integrity in a PI3K/Akt/mTOR‐dependent mechanism. Sci Rep. 2016; 6: 33636. 27644317 10.1038/srep33636 PMC5028890

[11] Zhou H , Qian J , Li C , *et al* Attenuation of cardiac dysfunction by HSPA12B in endotoxin‐induced sepsis in mice through a PI3K‐dependent mechanism. Cardiovasc Res. 2011; 89: 109–18. 20733008 10.1093/cvr/cvq268

[12] Li J , Zhang Y , Li C , *et al* HSPA12B attenuates cardiac dysfunction and remodelling after myocardial infarction through an eNOS‐dependent mechanism. Cardiovasc Res. 2013; 99: 674–84. 23729663 10.1093/cvr/cvt139

[13] Ma Y , Lu C , Li C , *et al* Overexpression of HSPA12B protects against cerebral ischemia/reperfusion injury *via* a PI3K/Akt‐dependent mechanism. Biochim Biophys Acta. 2013; 1832: 57–66. 23046810 10.1016/j.bbadis.2012.10.003

[14] Langhauser F , Kraft P , Gob E , *et al* Blocking of alpha4 integrin does not protect from acute ischemic stroke in mice. Stroke. 2014; 45: 1799–806. 24743435 10.1161/STROKEAHA.114.005000

[15] Zhao Y , Guan YF , Zhou XM , *et al* Regenerative neurogenesis after ischemic stroke promoted by nicotinamide phosphoribosyltransferase‐nicotinamide adenine dinucleotide cascade. Stroke. 2015; 46: 1966–74. 26060246 10.1161/STROKEAHA.115.009216

[16] Djakovic Z , Djakovic I , Cesarec V , *et al* Esophagogastric anastomosis in rats: improved healing by BPC 157 and L‐arginine, aggravated by L‐NAME. World J Gastroenterol. 2016; 22: 9127–40. 27895400 10.3748/wjg.v22.i41.9127 PMC5107594

[17] Wahl F , Allix M , Plotkine M , *et al* Neurological and behavioral outcomes of focal cerebral ischemia in rats. Stroke. 1992; 23: 267–72. 1561657 10.1161/01.str.23.2.267

[18] Han S , Tai C , Westenbroek RE , *et al* Autistic‐like behaviour in Scn1a+/‐ mice and rescue by enhanced GABA‐mediated neurotransmission. Nature. 2012; 489: 385–90. 22914087 10.1038/nature11356 PMC3448848

[19] Vahid‐Ansari F , Lagace DC , Albert PR . Persistent post‐stroke depression in mice following unilateral medial prefrontal cortical stroke. Transl Psychiatry. 2016; 6: e863. 27483381 10.1038/tp.2016.124 PMC5022078

[20] Thiyagarajan M , Fernandez JA , Lane SM , *et al* Activated protein C promotes neovascularization and neurogenesis in postischemic brain *via* protease‐activated receptor 1. J Neurosci. 2008; 28: 12788–97. 19036971 10.1523/JNEUROSCI.3485-08.2008 PMC2742231

[21] Wang Y , Kilic E , Kilic U , *et al* VEGF overexpression induces post‐ischaemic neuroprotection, but facilitates haemodynamic steal phenomena. Brain. 2005; 128: 52–63. 15509618 10.1093/brain/awh325

[22] Gray JD , Punsoni M , Tabori NE , *et al* Methylphenidate administration to juvenile rats alters brain areas involved in cognition, motivated behaviors, appetite, and stress. J Neurosci. 2007; 27: 7196–207. 17611273 10.1523/JNEUROSCI.0109-07.2007 PMC6794586

[23] Crawford CA , Der‐Ghazarian T , Britt CE , *et al* Novelty‐induced conditioned place preference, sucrose preference, and elevated plus maze behavior in adult rats after repeated exposure to methylphenidate during the preweanling period. Behav Brain Res. 2013; 246: 29–35. 23466690 10.1016/j.bbr.2013.02.031 PMC3636810

[24] Chen J , Zacharek A , Zhang C , *et al* Endothelial nitric oxide synthase regulates brain‐derived neurotrophic factor expression and neurogenesis after stroke in mice. J Neurosci. 2005; 25: 2366–75. 15745963 10.1523/JNEUROSCI.5071-04.2005 PMC2791344

[25] Santibanez JF , Letamendia A , Perez‐Barriocanal F , *et al* Endoglin increases eNOS expression by modulating Smad2 protein levels and Smad2‐dependent TGF‐beta signaling. J Cell Physiol. 2007; 210: 456–68. 17058229 10.1002/jcp.20878

[26] Chen L , Xiao J , Kuroda J , *et al* Both hydrogen peroxide and transforming growth factor beta 1 contribute to endothelial Nox4 mediated angiogenesis in endothelial Nox4 transgenic mouse lines. Biochim Biophys Acta. 2014; 1842: 2489–99. 25315297 10.1016/j.bbadis.2014.10.007

[27] He Y , Zhang H , Yung A , *et al* ALK5‐dependent TGF‐beta signaling is a major determinant of late‐stage adult neurogenesis. Nat Neurosci. 2014; 17: 943–52. 24859199 10.1038/nn.3732 PMC4096284

[28] Kazakov A , Hall R , Jagoda P , *et al* Inhibition of endothelial nitric oxide synthase induces and enhances myocardial fibrosis. Cardiovasc Res. 2013; 100: 211–21. 23863197 10.1093/cvr/cvt181

[29] Floel A , Cohen LG . Recovery of function in humans: cortical stimulation and pharmacological treatments after stroke. Neurobiol Dis. 2010; 37: 243–51. 19520165 10.1016/j.nbd.2009.05.027 PMC4886709

[30] Prakash R , Li W , Qu Z , *et al* Vascularization pattern after ischemic stroke is different in control versus diabetic rats: relevance to stroke recovery. Stroke. 2013; 44: 2875–82. 23920018 10.1161/STROKEAHA.113.001660 PMC3827629

[31] Ergul A , Alhusban A , Fagan SC . Angiogenesis: a harmonized target for recovery after stroke. Stroke. 2012; 43: 2270–4. 22618382 10.1161/STROKEAHA.111.642710 PMC3404267

[32] Ohab JJ , Fleming S , Blesch A , *et al* A neurovascular niche for neurogenesis after stroke. J Neurosci. 2006; 26: 13007–16. 17167090 10.1523/JNEUROSCI.4323-06.2006 PMC6674957

[33] Teng H , Zhang ZG , Wang L , *et al* Coupling of angiogenesis and neurogenesis in cultured endothelial cells and neural progenitor cells after stroke. J Cereb Blood Flow Metab. 2008; 28: 764–71. 17971789 10.1038/sj.jcbfm.9600573 PMC2744583

[34] Chen J , Cui X , Zacharek A , *et al* eNOS mediates TO90317 treatment‐induced angiogenesis and functional outcome after stroke in mice. Stroke. 2009; 40: 2532–8. 19443804 10.1161/STROKEAHA.108.545095 PMC2724074

[35] Luo Y , Zhao Y , Li X , *et al* ZNF580 mediates eNOS expression and endothelial cell migration/proliferation *via* the TGF‐beta1/ALK5/Smad2 pathway. Mol Cell Biochem. 2014; 393: 199–207. 24771066 10.1007/s11010-014-2061-z

[36] Halade GV , Ma Y , Ramirez TA , *et al* Reduced BDNF attenuates inflammation and angiogenesis to improve survival and cardiac function following myocardial infarction in mice. Am J Physiol Heart Circ Physiol. 2013; 305: H1830–42. 24142413 10.1152/ajpheart.00224.2013 PMC3882541

[37] Tonnessen BH , Severson SR , Hurt RD , *et al* Modulation of nitric‐oxide synthase by nicotine. J Pharmacol Exp Ther. 2000; 295: 601–6. 11046094

[38] Wang L , Kittaka M , Sun N , *et al* Chronic nicotine treatment enhances focal ischemic brain injury and depletes free pool of brain microvascular tissue plasminogen activator in rats. J Cereb Blood Flow Metab. 1997; 17: 136–46. 9040492 10.1097/00004647-199702000-00002

[39] Fujimura N , Jitsuiki D , Maruhashi T , *et al* Geranylgeranylacetone, heat shock protein 90/AMP‐activated protein kinase/endothelial nitric oxide synthase/nitric oxide pathway, and endothelial function in humans. Arterioscler Thromb Vasc Biol. 2012; 32: 153–60. 21998134 10.1161/ATVBAHA.111.237263

[40] Davis BJ , Xie Z , Viollet B , *et al* Activation of the AMP‐activated kinase by antidiabetes drug metformin stimulates nitric oxide synthesis *in vivo* by promoting the association of heat shock protein 90 and endothelial nitric oxide synthase. Diabetes. 2006; 55: 496–505. 16443786 10.2337/diabetes.55.02.06.db05-1064

[41] Selvaraju V , Suresh SC , Thirunavukkarasu M , *et al* Regulation of A‐kinase‐anchoring protein 12 by heat shock protein A12B to prevent ventricular dysfunction following acute myocardial infarction in diabetic rats. J Cardiovasc Transl Res. 2017; 10: 209–20. 28281242 10.1007/s12265-017-9734-4

